# Adolescent psychological stress impairs spermatogenesis by disrupting meiotic progression in male mice

**DOI:** 10.7717/peerj.21750

**Published:** 2026-10-02

**Authors:** Jiguang Gao, Wenwen Hou, Heli Wang, Shuo Xu, Guoxian Chen

**Affiliations:** 1 School of Basic Medical Sciences, Wannan Medical College, Wuhu, China; 2 Center of Reproductive Medicine, The First Affiliated Hospital of Wannan Medical College, Wuhu, China; 3 School of Clinical Medicine, Wannan Medical College, Wuhu, China

**Keywords:** Adolescence, Restraint stress, Spermatogenesis, Meiotic progression, Male reproductive toxicology

## Abstract

Psychological stress is a risk factor for male infertility, and adolescence is a critical period of vulnerability to stress. However, the effects of psychological stress on spermatogenesis in adolescent males remain unclear. To investigate this, we used a restraint stress model in adolescent Kunming mice, where animals were restrained for 2 and 4 h per day from postnatal day 28 to 56. Restraint stress resulted in a decreased sperm concentration and serum testosterone levels, together with increased sperm abnormalities, synaptonemal complex protein3 (SYCP3)-positive germ cells in the epididymis, and serum corticosterone levels in adult mice. Additionally, restraint stress altered seminiferous tubule histoarchitecture and the distribution of spermatogenic stages. Restraint-stressed mice exhibited increased proportions of zygotene- and pachytene-stage spermatocytes and a reduced proportion of diplotene-stage spermatocytes. Further analysis showed altered γH2AX and ataxia-telangiectasia and rad3-related (ATR) localization patterns together with increased frequencies of chromosomal synapsis abnormalities and abnormal chiasma formation, which were accompanied by increased spermatocyte apoptosis. The mRNA expression levels of key meiotic genes, including *SPO11 initiator of meiotic double stranded breaks* (*SPO11*), *DNA meiotic recombinase 1* (*DMC1*), *synaptonemal complex protein1* (*SYCP1*) and *SYCP3*, were reduced in stressed mice. In addition, restraint stress increased germ cell apoptosis and reduced litter size in adulthood. In conclusion, adolescent exposure to psychological stressors such as restraint stress is associated with impaired spermatogenesis and reduced reproductive capacity in adult male mice.

## Introduction

Psychological stress is recognized as an important risk factor for male reproductive disorders and infertility (Akram, Ali & Kaul, 2023; Gollenberg et al., 2010; Nargund, 2015). Adolescence represents a critical developmental period characterized by sensitivity and vulnerability to stress exposure (Crestani, 2017; Cruz et al., 2016). Evidence from rodent studies indicates that adolescents exhibit greater hypothalamic–pituitary–adrenal (HPA) axis activation and elevated plasma corticosterone responses to chronic stressors compared with adults (Duarte et al., 2015; Jankord et al., 2011). Dysregulation of the HPA axis can, in turn, suppress the hypothalamic–pituitary–gonadal axis, leading to reduced testosterone production and impaired spermatogenesis (Nargund, 2015). Despite the importance of adolescence as a key window for male reproductive development, the effects of psychological stress during this period have received far less attention than those during adulthood. Therefore, systematic investigation of how stress exposure during adolescence influences spermatogenesis and long-term male reproductive function is warranted.

Adolescence represents a critical window for male germ cell development, during which spermatogonia transition from mitotic proliferation to meiotic entry. Meiosis consists of two successive divisions, among which meiotic prophase I (MPI) is a particularly crucial and vulnerable stage in spermatogenesis. During normal meiosis, homologous chromosomes undergo pairing, synapsis, recombination, and accurate segregation (Jiang et al., 2018). Proper meiotic progression depends on the coordinated expression of multiple meiosis-related genes involved in DNA double-strand break formation, homologous recombination, and synaptonemal complex assembly. For example, *SPO11 initiator of meiotic double stranded breaks (SPO11)* recombination by inducing DNA double-strand breaks, *DNA meiotic recombinase 1 (DMC1)* mediates homologous recombination repair, while SYCP1 and SYCP3 are essential structural components of the synaptonemal complex required for homologous chromosome synapsis (Bolor et al., 2009). Disruption of these tightly regulated processes, including abnormal synaptonemal complex assembly or altered homologous recombination, has been associated with spermatocyte apoptosis and meiotic failure (Jiang et al., 2018; Bolor et al., 2009; Di Giacomo et al., 2005; Koskenniemi, Virtanen & Toppari, 2017). However, to what extent psychological stress–induced reproductive dysfunction interferes with meiotic progression during adolescence remains largely unclear.

Epidemiological studies have reported negative associations between perceived stress and exposure to multiple stressful life events and sperm concentration, motility, and morphology in adult men (Gollenberg et al., 2010; Janevic et al., 2014). Experimental studies further indicate that stressors impair testicular function and sperm quality in male rodents by reducing testosterone levels, increasing oxidative stress, and inducing germ cell apoptosis through activation of the TNF-α and FasL/Fas signaling pathways (Arun et al., 2016; Everds et al., 2013; Xiao et al., 2019; Yadav et al., 2022; Zhang et al., 2020). Collectively, these findings suggest that psychological and environmental stressors compromise male fertility by impairing sperm quality and promoting germ cell loss. However, the effects of psychological stress on spermatogenesis, with particular emphasis on meiotic processes, remain largely unexplored.

Restraint stress is one of the most commonly used experimental models of psychological stress in rodents (Bal et al., 2009; Glavin et al., 1994; Paré & Glavin, 1986). Although previous studies have demonstrated that stressors cause fertility decline in adult men and experimental animals, relatively few investigations have focused on the effects of psychological stress during adolescence on spermatogenesis. In the present study, we aim to characterize the impact of adolescent restraint stress on spermatogenesis in mice by evaluating sperm quality, testicular histology, and meiotic progression of spermatocytes.

## Materials and methods

### Experimental animals and design

SPF-grade male Kunming mice (postnatal day (PND) 21) were purchased from Hangzhou Medical College. All animals were specific pathogen-free (SPF) and had no known genetic modifications and were healthy at the start of the experiment. All animal procedures were approved by the Animal Ethics Committee of Wannan Medical College (Approval No. LLSC 2022-008) and were conducted in accordance with relevant institutional and national guidelines for the care and use of laboratory animals. Mice were housed in standard polypropylene cages (four per cage) under controlled environmental conditions (23 ± 2 °C, 50–70% relative humidity, 14 h light/10 h dark cycle) with free access to feed and water. Bedding material was changed regularly, and environmental enrichment (*e.g*., nesting material) was provided to promote natural behavior and welfare. After 1 week of acclimatization, a total of 24 mice were randomly assigned to three groups (*n* = 8 per group): restraint stress control (RSC), restraint stress for 2 h/day (RS2), and restraint stress for 4 h/day (RS4). The sample size was chosen based on previous studies using similar mouse models, in which this sample size was sufficient to detect significant differences in the primary outcome measure (*e.g*., sperm concentration and sperm morphology). No formal *a priori* sample size calculation was performed. For restraint stress, mice were individually placed in a well-ventilated steel-wire mesh cage that allowed limited forward and backward movement but prevented turning around (Deng et al., 2021; Xu et al., 2020; Zhang et al., 2011). Restraint was applied for 2 or 4 h per day for 4 consecutive weeks. During the restraint period, mice had no access to food or water. All experimental procedures, including restraint stress and sample collection, were performed at consistent times of day using standardized protocols. Animals showing signs of illness or abnormal behavior during acclimation were excluded from the study. No animals died or were excluded during the restraint stress procedures.

Animals were monitored daily for general health status, including body weight, activity, grooming behavior, and signs of distress. Humane endpoints were predefined, and animals showing severe weight loss (>20% of initial body weight), persistent lethargy, or signs of severe distress were to be euthanized prior to the planned endpoint; however, no animals met these criteria during the study. At the end of the experimental period, mice were euthanized by cervical dislocation performed by trained personnel in accordance with institutional guidelines, and samples were collected for subsequent analyses.

### Body weight gain and reproductive organs index

All mice were euthanized on PND 57, and reproductive organs, including the testes, epididymides, and seminal vesicles, were collected and weighed. Relative reproductive organ weight was calculated as organ weight normalized to body weight and expressed as organ weight per 100 g body weight. Body weight was recorded daily throughout the experimental period, and body weight gain was calculated as percentage increase relative to the initial body weight.

### Histological studies of reproductive organs

The testis and epididymis were fixed in 4% paraformaldehyde for 12 h and embedded in paraffin, sectioned at 5 μm and stained with hematoxylin and eosin (H&E) for histological evaluation (Zhang et al., 2010). Germ cell apoptosis in the testes was assessed using a TUNEL assay kit (MK1011-50; Boster Biological Technology, Wuhan, China) according to the manufacturer’s instruction. TUNEL-positive cells were identified by brown nuclear staining. For each animal, at least 30 randomly selected seminiferous tubules were analyzed. The apoptotic rate was expressed as the percentage of seminiferous tubules containing TUNEL-positive cells among the total seminiferous tubules analyzed.

Testicular sections were further subjected to Periodic Acid–Schiff (PAS) staining to determine the stages of the seminiferous epithelium cycle, as previously described (Hess & Renato de Franca, 2008). For quantitative analysis, adjacent seminiferous epithelial stages with similar cellular associations were grouped to improve consistency of stage identification and statistical analysis. Degenerating germ cells (or degenerative spermatid-like cells) were identified based on nuclear pyknosis, cytoplasmic shrinkage, and detachment from the seminiferous epithelium. Immunofluorescence staining of epididymal sections was performed as previously described (Gao et al., 2019). Finally, all sections were examined using a Nikon fluorescence microscope (Ni-E; Nikon, Tokyo, Japan). Histological evaluations were performed in a blind manner. At least 30 seminiferous tubule cross-sections per animal were examined for stage determination.

### Measurement of serum corticosterone and testosterone

Blood samples were collected from mice at PND 57 immediately after sacrifice. To minimize circadian variation, all samples were obtained between 09:00 and 11:00 a.m. After standing at room temperature for 30 min, the blood samples were centrifuged at 3,000 g for 15 min at 4 °C to obtain serum. Serum corticosterone and testosterone concentrations were measured using commercially available enzyme-linked immunosorbent assay (ELISA) kits (corticosterone: JYM0544Mo; JYMbio, Wuhan, China; testosterone: JYM0373Mo; JYMbio, Wuhan, China), according to the manufacturers’ instructions. All samples were assayed in duplicate. Hormone concentrations were calculated based on standard curves generated for each assay.

### Assessment of male reproductive parameters

Sperm counting and morphological analyses were described in our previous article (Gao et al., 2022). Briefly, the cauda epididymis was unilaterally incised in 2 mL F12 medium for 30 min. The sperm concentration was measured using a hemocytometer. For sperm morphology analyses, sperm were smeared on slides, fixed in methanol, and stained with 0.25% eosin stain for 1 h. Slides were examined under a microscope (Ni-E; Nikon, Tokyo, Japan). For each animal, at least 100 spermatozoa were examined under light microscopy.

Male fertility was assessed at the end of the restraint stress protocol. Adult male mice (PND 56) were co-housed overnight with two sexually mature untreated female mice (8–10 weeks old) at a ratio of 1:2. The estrous cycle stage of females was determined by vaginal cytology, and only females in proestrus were used for mating. Successful copulation was confirmed by the presence of a vaginal plug the following morning. Females with a vaginal plug were maintained individually until delivery, and litter size was recorded at birth. Male mice were euthanized on PND 57 for tissue collection and subsequent analyses.

### Meiotic spreads assays

Meiotic spreads and immunofluorescence staining were performed according to previous methods (Gely-Pernot et al., 2015). Testes were chopped and filtered in PBS. The cell suspension was centrifuged, resuspended in 0.5% NaCl for 5 min, and smeared onto slides. After drying, the slides were fixed with 1~2% paraformaldehyde. The fixed slides were blocked with 5% bovine serum albumin (BSA) for 1 h, incubation of primary (anti-SYCP3: SC-74569; Santa Cruz Biotechnology, Dallas, TX, USA; anti-γH2AX: 9718S; Cell Signaling Technology, Danvers, MA, USA; anti-SYCP1: AB15090; Abcam, Cambridge, UK; ataxia-telangiectasia and rad3-related (ATR): 19787-1-AP; Proteintech, Rosemont, IL, USA) and secondary antibodies (Dylight 488 conjugated goat anti-rabbit IgG secondary antibody: A23220; Abbkine, Atlanta, GA, USA; Dylight 549 conjugated goat anti-mouse IgG secondary antibody: A23310; Abbkine, Atlanta, GA, USA). Slides were examined by Nikon fluorescence microscope (Ni-E; Nikon, Tokyo, Japan). The meiotic substages were determined based on the previously described methods (Gao et al., 2022; Gely-Pernot et al., 2015). Pachytene spermatocytes showing γH2AX or ATR signals extending beyond the sex body and distributed along autosomes were defined as exhibiting abnormal signal patterns. Incomplete overlap or discontinuous alignment of homologous chromosomes along the synaptonemal complex was considered abnormal synapsis.

### Ribonucleic acid extraction and cDNA synthesis

The testicular tissue was snap-frozen in liquid nitrogen and stored at −80 °C. Total ribonucleic acid (RNA) was extracted from adult mouse testes (50 mg/sample) using TRIzol reagent (R0016; Beyotime, Shanghai, China) according to the manufacturer’s instructions. RNA concentration and purity were assessed using a NanoDrop 2000 spectrophotometer (Thermo Fisher Scientific, Waltham, MA, USA). Because all RNA samples exhibited A260/A280 ratios between 1.8 and 2.0, they were considered suitable for downstream analysis. All RNA samples were treated with DNase I (2270A; Takara, Tokyo, Japan) prior to cDNA synthesis to remove potential genomic DNA contamination. For cDNA synthesis, 1 µg of total RNA was reverse transcribed using the PrimeScript RT Reagent Kit (MT04100; Beyotime, Shanghai, China) in a 20 µL reaction, containing 4 µL 5× PrimeScript Buffer, 1 µL PrimeScript RT Enzyme Mix I, one µL Random Primer Mix, and nuclease-free water. The reverse transcription reaction was performed at 37 °C for 15 min, followed by 85 °C for 5 s to inactivate the enzyme. The cDNA was stored at –20 °C for subsequent analysis.

### Quantitative PCR

Quantitative PCR (qPCR) was performed using the SYBR Green Premix (D7260; Beyotime, Shanghai, China) on an Applied Biosystems 7500 Real-Time PCR System (Thermo Fisher Scientific, Waltham, MA, USA). Each 20 µL reaction contained 10 µL SYBR Green Premix, 0.4 µL of each forward and reverse primer (final concentration 200 nM), two µL cDNA template (corresponding to 100 ng RNA input), and 7.2 µL nuclease-free water. The cycling conditions were: initial denaturation at 95 °C for 30 s, followed by 40 cycles of 95 °C for 5 s and 60 °C for 30 s. A melt curve analysis (65–95 °C, 0.5 °C increments) was performed to confirm the specificity of amplification. All reactions were performed in technical triplicates, with three biological replicates per group. At low template concentrations, the Cq variation among technical replicates was ≤0.5 cycles, demonstrating reproducible amplification at the detection limit. No amplification was observed in no-template controls. The relative gene expression was calculated using the 2^−ΔΔCt^ method, with Actb used as the reference gene. The forward and reverse primer sequences, amplicon sizes are provided in Table S1, following the Minimum Information for Publication of Quantitative Real-Time PCR Experiments (MIQE) recommendations.

### Data analysis

The data were expressed as mean ± standard error of the mean (SEM). All data met the assumptions for parametric testing. Differences among study groups were analyzed using one-way analysis of variance (ANOVA) followed by Tukey’s *post hoc* test. Statistical analysis were performed using SPSS 20 (IBM Co., Armonk, NY, USA). Graphs were plotted in Prism V8.0.2.263 (GraphPad software Inc., La Jolla, CA, USA). *P* < 0.05 was considered statistically significant. Group allocation was known to the investigators during animal assignment and the restraint stress procedures. Outcome assessments, including histological evaluation and sperm analysis, were performed by investigators blinded to group allocation. Data analysis was conducted using coded datasets to minimize potential bias.

## Results

### Adolescent restraint stress alters body weight, reproductive organ weights, and serum hormone levels in adult mice

Compared with the RSC group, body weight gain expressed as a percentage of initial body weight was significantly reduced in restraint-stressed groups (Fig. 1A). Analysis of relative reproductive organ weight (mg/100 g BW) revealed significant reductions in testes and seminal vesicles in the RS4 group (Figs. 1B–1E), whereas the relative weight of the epididymis did not differ significantly among groups. While some parameters in the RS2 group showed a decreasing trend compared with the RSC group, all changes were more pronounced in the RS4 group, indicating a duration-dependent effect. Consistent with the physiological stress response, serum corticosterone levels were significantly increased in restraint-stressed mice (Fig. 1F), whereas serum testosterone levels were significantly reduced (Fig. 1G). Overall, adolescent restraint stress was associated with reduced reproductive organ weight and altered hormone levels in adult mice.

**Figure 1:**
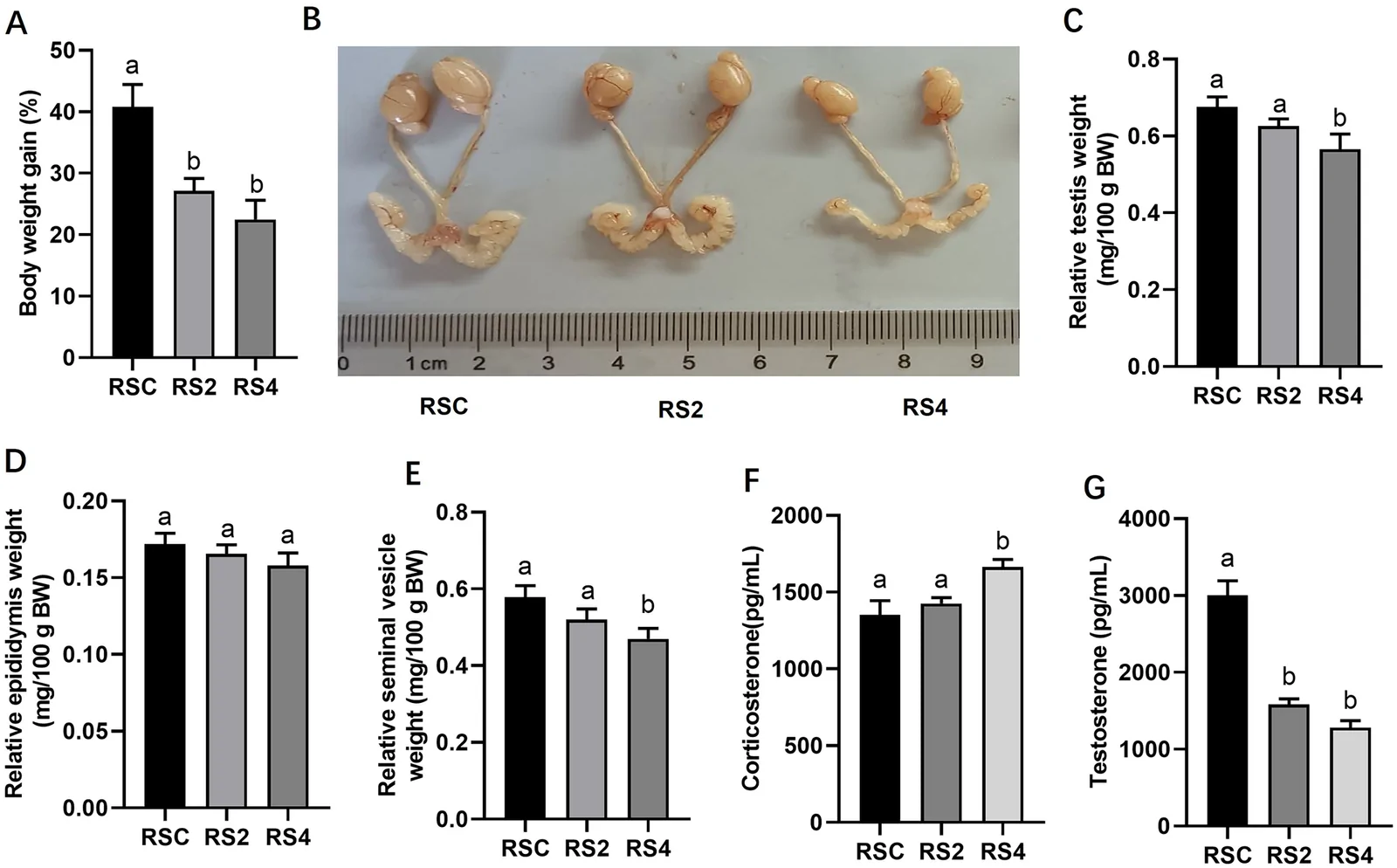
Effects of restraint stress on body weight gain, relative reproductive organ weights, and serum corticosterone and testosterone levels in adult mice. (A) Body weight gain expressed as percentage of initial body weight in control and restraint-stressed mice. (B) The overall size of reproductive organs. (C–E) Relative weights of testes, epididymides, and seminal vesicles (mg/100 g body weight). (F) Serum corticosterone levels in control and restraint-stressed mice. (G) Serum testosterone levels in control and restraint-stressed mice. a and b indicate significant differences among different groups (*P* < 0.05).

### Adolescent restraint stress impairs testis histology and sperm quality in adulthood

Compared to the RSC group, testicular histoarchitecture was significantly impaired in restraint-stressed mice (Fig. 2A). The germinal epithelium and germ cells appeared loose and vacuolized in both RS groups, with a duration-dependent decrease in the seminiferous tubule area (Fig. 2B). Degenerating germ cell-like structures were predominantly observed in the caput and cauda of the epididymis (Fig. 2A), whereas no obvious histopathological alterations were observed in the epididymal epithelium. Sperm concentration in the cauda epididymis was significantly reduced (Fig. 2C), and the proportion of abnormal sperm was significantly increased following restraint stress (Figs. 2D and 2E). Together, these results demonstrate histopathological alterations in the testis and increased abnormal spermatozoa following adolescent restraint stress.

**Figure 2:**
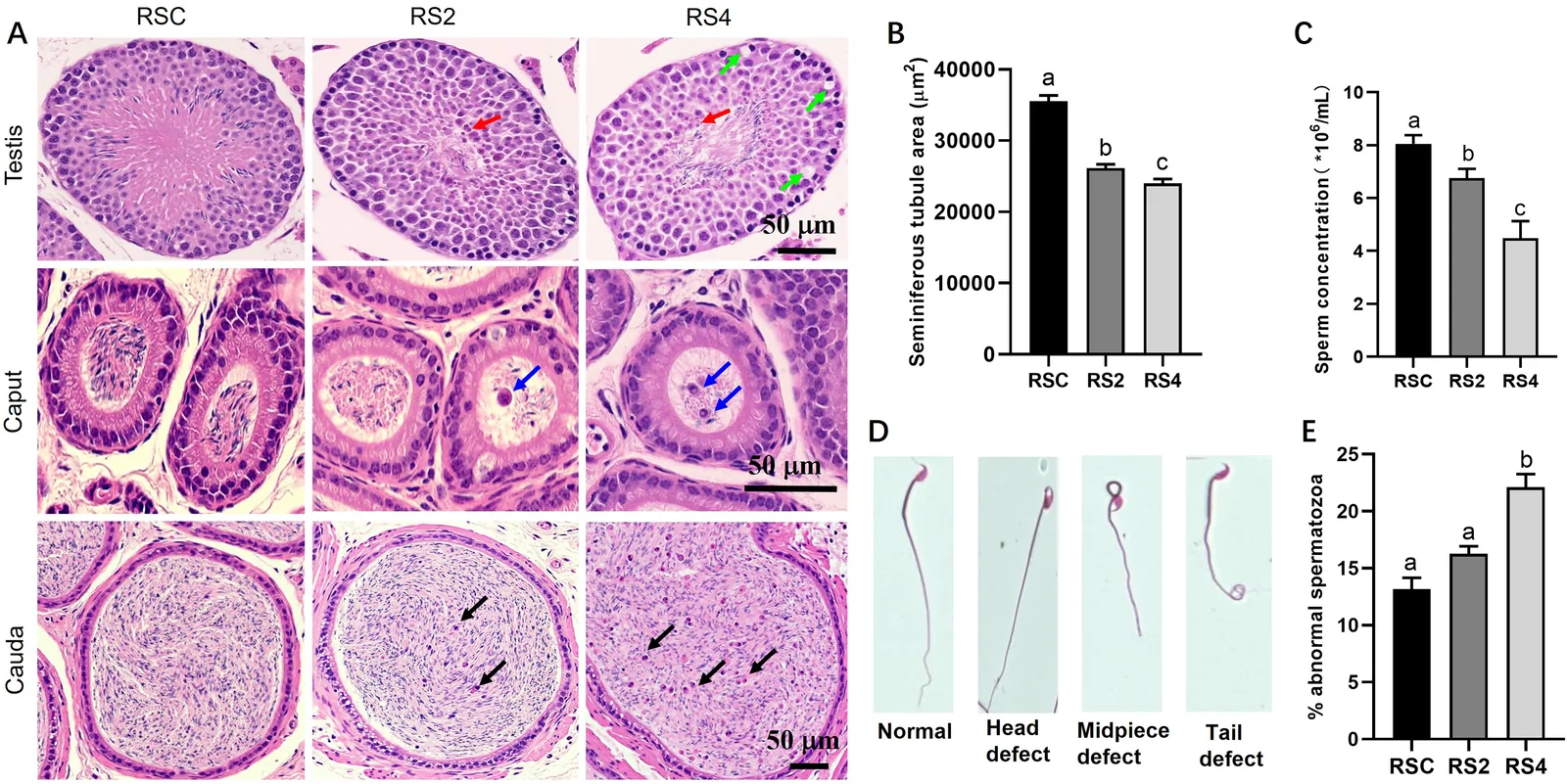
Effects of restraint stress on testicular and epididymal histology, sperm concentration and morphology in mice. (A) Representative H&E staining of testes, caput epididymis and cauda epididymis. Red arrows: degenerating germ cell-like structures in seminiferous tubule lumen; green arrows: intraepithelial vacuoles; blue arrows: degenerating germ cell-like structures in caput epididymis; black arrows: degenerating germ cell-like structures in cauda epididymis. (B) Average area of seminiferous tubules in RSC and restraint-stressed mice (μm^2^). (C) Sperm concentration in the unilateral cauda epididymis. (D) Representative images of morphologically abnormal sperm. (E) Percentage of abnormal spermatozoa in the cauda epididymis. Scale bars = 50 μm. a, b, and c indicate significant differences among different groups (*P* < 0.05).

### Restraint stress alters spermatogenic stage distribution in seminiferous tubules

To explore the effects of restraint stress on spermatogenesis, histopathological changes in the seminiferous epithelium were evaluated in adult mice (Fig. 3A). Degenerating germ cell-like cells were frequently observed within seminiferous tubules of restraint-stressed mice, whereas such structures were rarely observed in control mice (Fig. 3A). Quantitative analysis demonstrated that restraint stress altered the distribution of seminiferous epithelial stages (Fig. 3B). Compared with the control group, the proportion of seminiferous tubules at stages I–VI was significantly reduced, whereas the proportion at stages IX–XII was significantly increased in restraint-stressed mice. No significant differences were observed in the proportion of seminiferous tubules at stages VII–VIII between groups (Fig. 3B). Furthermore, primary spermatocytes, identified by immunostaining for the synaptonemal complex protein SYCP3, were detected in the epididymis of restraint-stressed mice (Fig. 3C). Collectively, these findings were accompanied by the accumulation of late-stage spermatocytes and the ectopic presence of SYCP3-positive cells in the epididymis.

**Figure 3:**
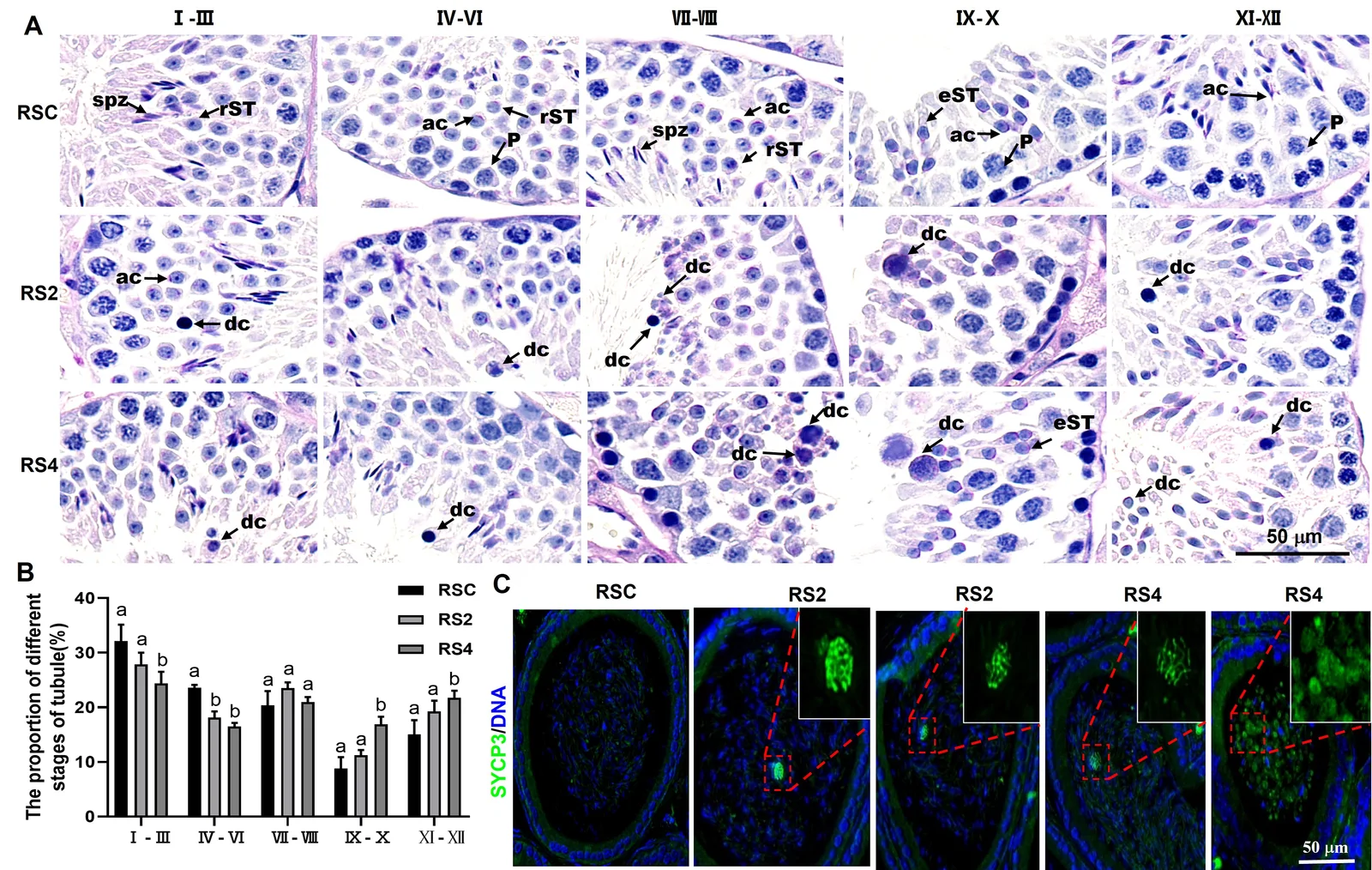
Effects of restraint stress on the spermatogenic stages in adult mice. (A) Representative periodic acid-Schiff (PAS) and hematoxylin staining of testes. P, pachytene spermatocyte; rST, round spermatid; eST, elongating spermatid; M, meiotic spermatocyte; spz, spermatozoa; ac, acrosome; dc, degenerating germ cell-like structures. (B) Distribution of seminiferous tubule cross-sections at different stages of the spermatogenic cycle in RSC and restraint-stressed mice. (C) Immunofluorescence staining of SYCP3 (green), a meiotic spermatocyte marker, in the epididymis of RSC and restraint-stressed mice. Scale bars = 50 μm. a and b indicate significant differences among different groups (*P* < 0.05).

### Restraint stress alters meiotic progression in primary spermatocytes

To further investigate whether restraint stress was associated with alterations in meiotic progression, primary spermatocytes were analyzed in adult mice (Fig. 4A). Quantitative analysis revealed a significant increase in the proportion of spermatocytes at the zygotene stage, accompanied by a marked reduction in diplotene-stage spermatocytes in restraint-stressed mice (Fig. 4B). In addition, the percentages of spermatocytes at the zygotene and pachytene stages were elevated in the stressed groups compared with the RSC group. These alterations in spermatocyte stage distribution were accompanied by reduced mRNA expression levels of several meiosis-related genes, including *SPO11*, *DMC1*, *SYCP1*, and *SYCP3* (Figs. 4C–4F). Overall, restraint stress was associated with an increased proportion of spermatocytes at the zygotene and pachytene stages.

**Figure 4:**
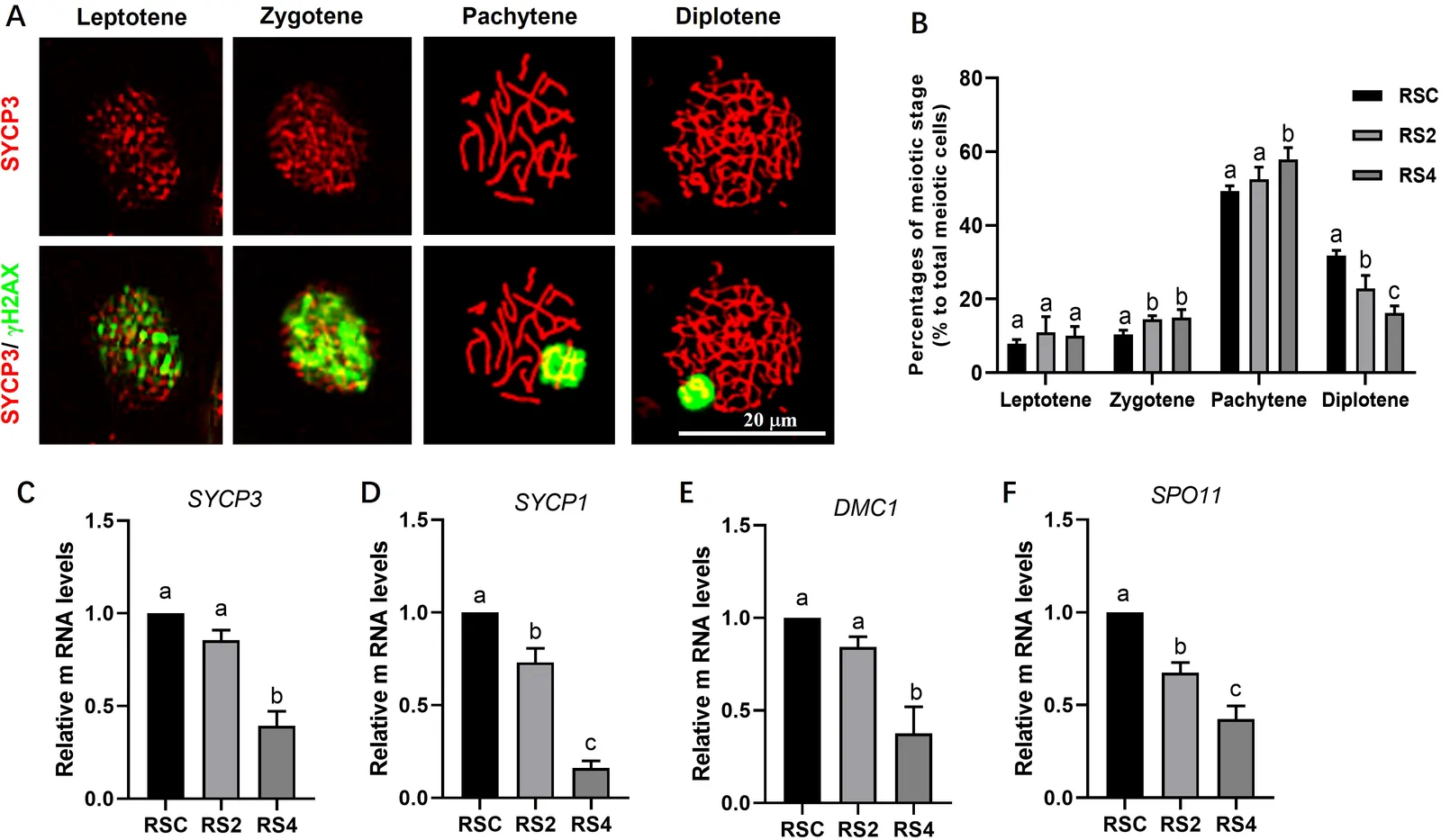
Effects of restraint stress on meiotic progression in spermatocytes of adult mice. (A) Representative immunofluorescence images for SYCP3 (red) and γH2AX (green) in spermatocytes at different sub-stages of meiotic prophase I. (B) The proportion of spermatocytes at the four meiotic sub-stages. (C–F) Relative expression of meiosis-related genes involved in DNA double-strand break formation (*SPO11*), homologous recombination (*DMC1*), and synaptonemal complex structure (*SYCP3*, *SYCP1*) was determined by qRT-PCR. More than 150 spermatocytes were classified per group. Scale bars = 20 μm. a, b, and c indicate significant differences among different groups (*P* < 0.05).

### Restraint stress alters ATR and γH2AX distribution patterns in pachytene-stage spermatocytes

The formation and repair of programmed double-strand breaks are essential events during meiotic prophase I. To investigate whether restraint stress affected the distribution of ATR and γH2AX in pachytene-stage spermatocytes, we analyzed their localization patterns. Abnormal spermatocytes showed ATR and γH2AX signals extending to autosomal regions beyond the sex body. In control mice, ATR and γH2AX signals were predominantly restricted to the sex chromosome region, forming a distinct sex body in pachytene spermatocytes (Figs. 5A and 5C). In contrast, in restraint-stressed mice, aberrant ATR and γH2AX signals were observed not only on the sex chromosomes but also ectopically distributed along the autosomes in a subset of pachytene spermatocytes (Figs. 5A and 5C). Quantitative analysis revealed that the proportion of pachytene spermatocytes exhibiting abnormal ATR and γH2AX distribution patterns was significantly increased in the RS4 group compared with the RSC group (Figs. 5B and 5D). ATR and γH2AX normally mark sites of DNA double-strand breaks and participate in the DNA damage response during meiosis, and their abnormal localization to autosomes is consistent with the known meiotic silencing of unsynapsed chromatin (MSUC) mechanism (Fernández et al., 2019). Together, these results demonstrate altered ATR and γH2AX distribution patterns in pachytene-stage spermatocytes following restraint stress.

**Figure 5:**
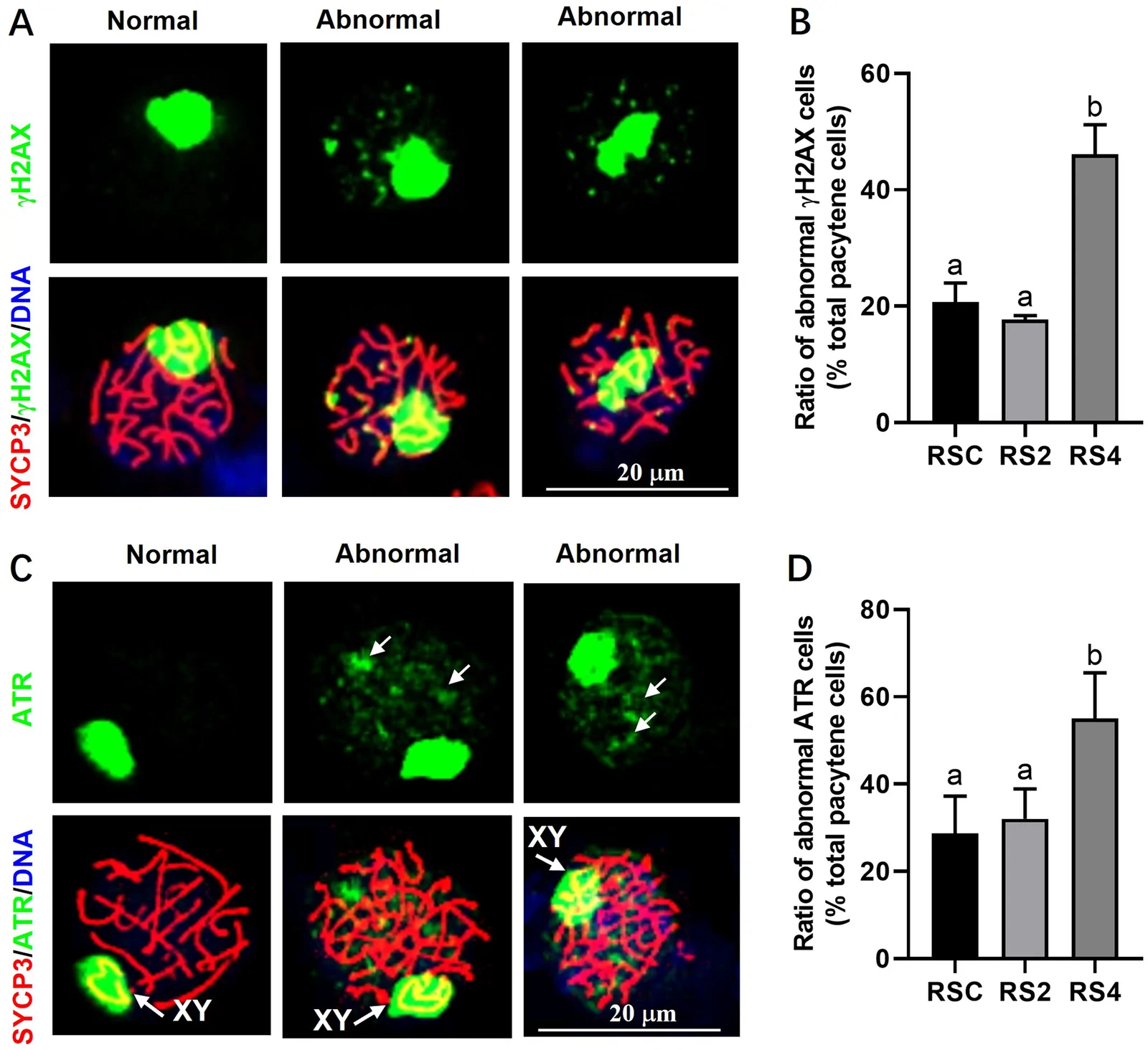
Effects of restraint stress on γH2AX and ATR distribution patterns in pachytene-stage spermatocytes. (A) Representative immunofluorescence images of normal and abnormal γH2AX (green) distribution patterns in pachytene-stage spermatocytes. (B) Quantification of the proportion of pachytene-stage spermatocytes exhibiting abnormal γH2AX distribution patterns. (C) Representative immunofluorescence images of normal and abnormal ATR (green) distribution patterns in pachytene-stage spermatocytes. (D) Quantification of the proportion of pachytene-stage spermatocytes exhibiting abnormal ATR distribution patterns. More than 300 spermatocytes were classified per group. Scale bars = 20 μm. a and b indicate significant differences among different groups (*P* < 0.05).

### Restraint stress impairs homologous chromosome synapsis and chiasma formation in spermatocytes

To determine whether homologous chromosomal synapsis was affected by restraint stress, synapsis and chiasma formation along the synaptonemal complex were examined in spermatocytes. In the RS4 group, 54.97% of homologous chromosomes exhibited incomplete overlap along the synaptonemal complex, which was significantly higher than the 20.51% observed in the RSC group (Figs. 6A and 6B).

**Figure 6:**
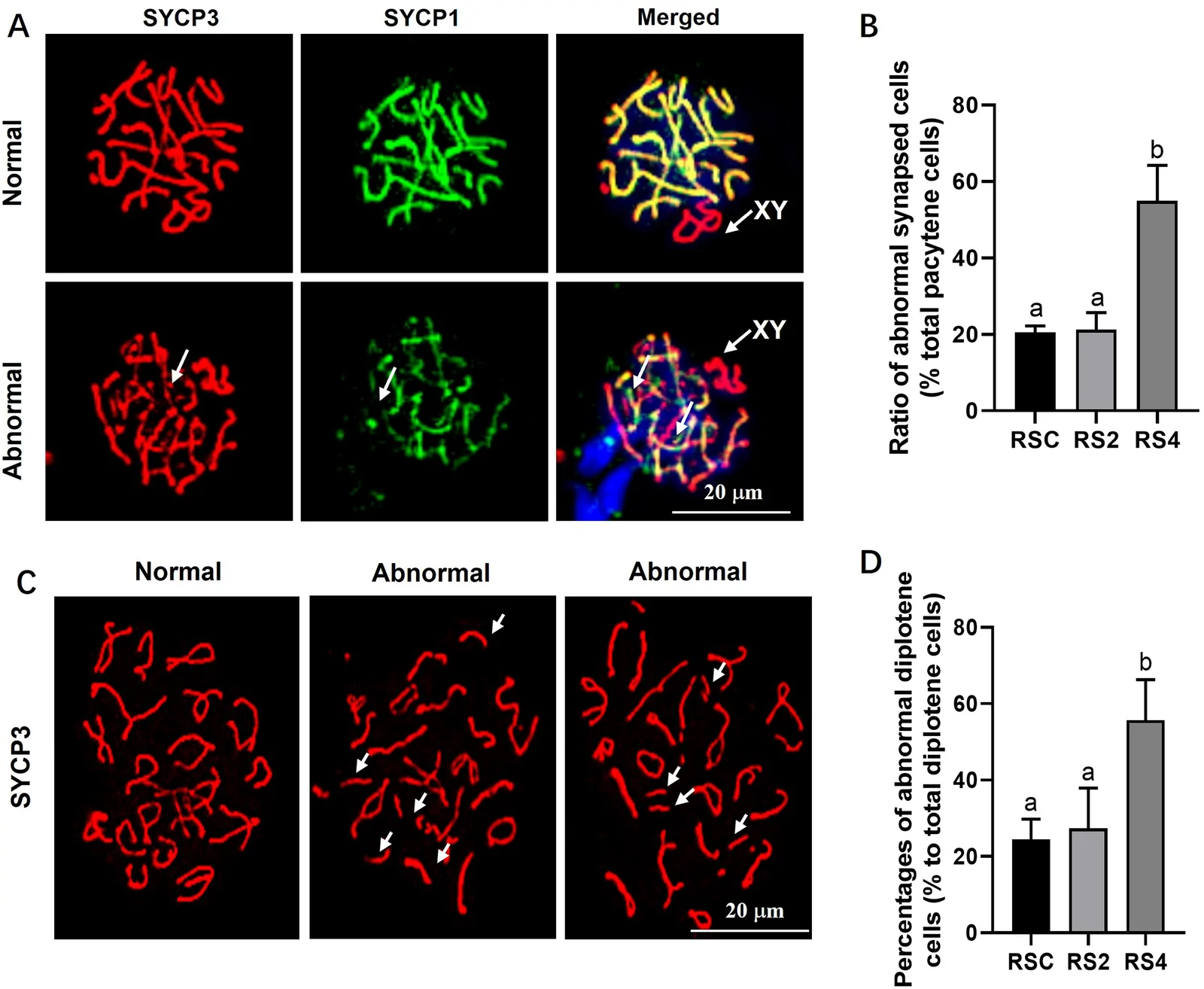
Effects of restraint stress on homologous chromosome synapsis and chiasma formation in spermatocytes of adult mice. (A) Representative immunofluorescence images of SYCP1 (green) and SYCP3 (red) in pachytene-stage spermatocytes. Arrows indicate incompletely synapsed homologous chromosomes. (B) The proportion of abnormal synapsed chromosomes in pachytene-stage spermatocytes. (C) Representative immunofluorescence images of SYCP3 (red) in late diplotene-stage spermatocytes. Arrows indicate univalent chromosomes. (D) Quantification of abnormal separated homologous chromosomes in late diplotene-stage spermatocytes. More than 300 spermatocytes were classified per group. Scale bars = 20 μm. a and b indicate significant differences among different groups (*P* < 0.05).

As meiosis progresses, crossovers formed between homologous chromosomes during pachytene are converted into chiasmata in late diplotene spermatocytes. Therefore, chiasma formation was further assessed in diplotene spermatocytes using SYCP3 staining of chromosome surface spreads (Fig. 6C). In the RSC group, homologous chromosomes were predominantly observed as de-synapsed bivalents with intact chiasmata (Fig. 6C). In contrast, in both the RS2 and RS4 groups, at least one pair of univalent was frequently observed (Fig. 6C). The proportion of late diplotene spermatocytes exhibiting abnormal chiasma formation was significantly higher in the RS4 group compared with the RSC group (Fig. 6D). Collectively, these findings show increased frequencies of chromosomal synapsis abnormalities and abnormal chiasma patterns in restraint-stressed mice.

### Adolescent restraint stress increases spermatocyte apoptosis and reduces litter size in adult mice

To determine whether restraint stress induced spermatocyte apoptosis in the testis, apoptotic cells were evaluated by TUNEL staining (Turner, 2007). Compared with the RSC group, the proportion of seminiferous tubules containing TUNEL-positive cells was markedly increased in restraint-stressed mice. Notably, TUNEL-positive signals were predominantly observed in pachytene-stage spermatocytes within the seminiferous epithelium (Figs. 7A and 7B). To further evaluate the impact of restraint stress on litter size, adult control and restraint-stressed male mice were mated with untreated females. Males exposed to restraint stress during adolescence exhibited a significant reduction in litter size compared with control males, particularly in the RS4 group.

**Figure 7:**
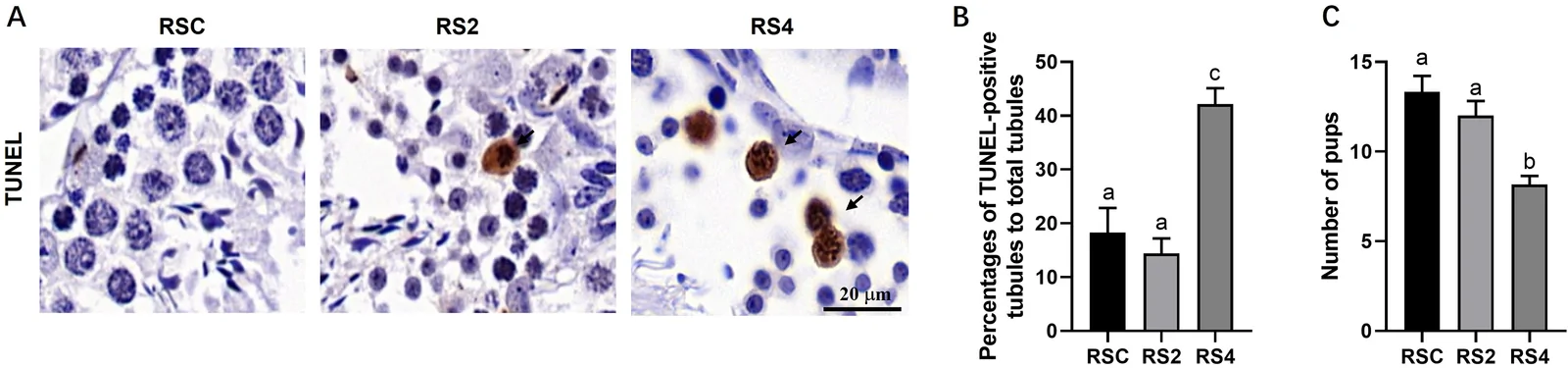
Effects of restraint stress on germ cell apoptosis and male fertility in adult mice. (A) Representative TUNEL staining images of testes. Arrows indicate apoptotic pachytene-stage spermatocytes. (B) The proportion of TUNEL-positive seminiferous tubules relative to the total number of tubules. (C) Litter size of untreated females mated with control and restraint-stressed male mice. Scale bars = 20 μm. a, b and c indicate significant differences among different groups (*P* < 0.05).

## Discussion

Restraint is a widely used experimental paradigm for modeling psychogenic stress (Glavin et al., 1994; Paré & Glavin, 1986). To investigate the effects of psychological stress (restraint stress) on spermatogenesis, adolescent male mice were subjected to restraint stress for 2 or 4 h per day from PND 28 to 56. In many restraint stress protocols, mice are confined in 50 mL centrifuge tubes or metal tubes (Deng et al., 2021; MacNiven, deCatanzaro & Younglai, 1992); however, such devices can impair heat dissipation and elevate the ambient temperature within the restraint apparatus. To minimize potential confounding effects of temperature, mice in the present study were individually restrained in small steel-wire mesh cages (Xiao et al., 2019; Zhang et al., 2011). Using this refined restraint model, we found that adolescent restraint stress was associated with reduced sperm quality, alterations in spermatogenesis and meiotic progression in adult mice.

### Effects of restraint stress on body weight, testicular structure, and sperm quality

Adolescence represents a critical period for somatic growth and reproductive system maturation, during which body weight gain and reproductive organ development serve as important indicators of normal development. Previous studies have revealed that prolonged restraint stress (3~4 h per day for 30~42 days) significantly reduces body weight gain and testis weight in adult rats (Arun et al., 2016; Yadav et al., 2022). Similarly, reduced body weight gain has been observed in adolescent rats following daily restraint stress for 1 h over 13 days (Xu et al., 2020). Consistent with these findings, our study demonstrates that restraint stress during adolescence significantly impairs body weight gain and reduces reproductive organ weights in mice.

Restraint stress is known to activate the hypothalamic–pituitary–adrenal axis, resulting in elevated corticosterone levels and reduced testosterone production (Arun et al., 2016; MacNiven, deCatanzaro & Younglai, 1992; Zardooz et al., 2006). In addition, restraint stress increases energy expenditure without compensatory increases in food intake (Bhatnagar et al., 2006; Yam et al., 2017). Chronic stress has also been reported to alter appetite-regulating hormones and neuropeptides, including leptin, ghrelin, and neuropeptide Y, which may influence energy balance and body weight regulation (Harris et al., 1998). In the present study, restraint-stressed mice exhibited elevated corticosterone levels together with reduced testosterone levels, consistent with previous psychological stress models and supporting the effectiveness of the restraint protocol. These endocrine and metabolic alterations may contribute to the reduced body weight gain and relative reproductive organ weights observed in adult mice following adolescent restraint stress.

Histological analyses further revealed that restraint stress compromised testicular structural integrity, as evidenced by increased degenerating germ cell-like structures and disorganization of the germinal epithelium within the seminiferous tubules. These pathological alterations are consistent with those reported in adult male mice subjected to restraint stress (Arun et al., 2016; Mehfooz et al., 2018; Priya & Reddy, 2012). Moreover, degenerating germ cell-like structures were frequently observed in the epididymis of restraint-stressed mice, consistent with the reduced sperm concentration and increased sperm abnormalities observed in these animals.

Sperm concentration, morphology, and viability are critical indicators of male reproductive health. Previous studies have shown that restraint stress reduces sperm count and increases sperm abnormalities in adult male rodents (Yadav et al., 2022; Hirano et al., 2014; Mustafa et al., 2020). Prolonged restraint stress has also been reported to induce sperm mitochondrial dysfunction, trigger germ cell apoptosis, and activate the TNF-α/FasL signaling pathway (Xiao et al., 2019; Zhang et al., 2020), as well as impair acrosome reactions and reduce *in vitro* fertilization rates (Arun et al., 2016; Zhang et al., 2020). Consistent with previous studies, restraint-stressed mice exhibited reduced sperm concentration and increased sperm abnormalities in adult male mice. In addition, sperm motility and viability were not evaluated in the present study, which may limit the comprehensive assessment of sperm function following restraint stress. Taken together, these findings indicate that adolescent restraint stress is associated with impaired reproductive organ development, histopathological alterations, and reduced sperm quality in adulthood.

### Effects of restraint stress on the spermatogenesis cycle and meiotic progression of spermatocytes

Spermatogenesis is a highly ordered process comprising mitotic proliferation, meiotic division, and spermiogenesis (de Kretser et al., 1998; Ishiguro, 2024; Jan et al., 2012). In the present study, adolescent restraint stress increased the proportion of abnormal seminiferous tubules and altered spermatogenic stage distribution in adult mice. The spermatogenic cycle encompasses successive phases of germ cell proliferation, differentiation, meiosis, and spermatid morphological transformation (Jan et al., 2012; de Rooij, 2001). In mice, seminiferous tubules are classified into 12 stages based on the differentiation status of spermatogenic cells (Meistrich & Hess, 2013; Oakberg, 1956). In the present study, restraint stress altered the distribution of seminiferous epithelial stages, characterized by an accumulation of seminiferous tubules at stages IX–XII and a reduction at stages I–VI, reflecting an altered distribution of spermatogenic stages rather than evidence of complete spermatogenic arrest. Stages IX–XII represent a critical transition period characterized by meiotic divisions of spermatocytes and the initiation of spermatid elongation (Wakayama et al., 2022). Although stages VII–VIII were not significantly altered, the observed alterations in other stages indicate changes in spermatogenic stage distribution that may contribute to reduced sperm concentration, without implying direct impairment of testosterone-dependent stages. Notably, an increased proportion of pachytene- or diplotene-stage spermatocytes was observed at stage XII in restraint-stressed mice. In addition, degenerating spermatid-like cells were observed within the seminiferous tubule lumen, and pachytene-stage spermatocytes were detected in the epididymis, further supporting an association with altered meiotic progression.

Meiosis is an essential process in spermatogenesis, and any mistake during this process may lead to failure of spermatogenesis (Ishiguro, 2024; Cooke & Saunders, 2002). Pachytene-stage spermatocytes in restraint-stressed mice exhibited persistent γH2AX and ATR signals on autosomes, whereas in control mice these markers are normally confined to the sex chromosomes. Meiotic prophase I is characterized by programmed DNA double-strand break formation and repair, processes that are tightly regulated by the expansion of phosphorylated H2AX and the recruitment of DNA damage response factors such as DMC1 and ATR (Jiang et al., 2018; Li et al., 2021). Abnormal localization of these proteins to autosomal regions may reflect defective DSB repair and activation of MSUC, a mechanism that silences transcription from unsynapsed autosomes (Fernández et al., 2019; Longhese et al., 2009). Disrupted DSB repair on autosomes can compromise homologous chromosome synapsis, recombination, and crossover formation, consistent with the increased incidence of incompletely synapsed and fully de-synapsed bivalents observed in stressed mice. In the present study, γH2AX and ATR signals persisted on autosomes in pachytene-stage spermatocytes following restraint stress, suggesting persistent DNA damage and defective DNA double-strand break repair. Persistent γH2AX signaling has also been linked to pachytene arrest and apoptosis of spermatocytes, which may be associated with the increased frequency of apoptotic germ cells observed in stressed mice.

In addition, key meiosis-related genes, including *SPO11*, *DMC1*, *SYCP1*, and *SYCP3*, were downregulated in restraint-stressed mice. *SPO11* is responsible for initiating DSBs, *DMC1* mediates strand exchange during homologous recombination, and *SYCP1* and *SYCP3* are structural components of the synaptonemal complex required for chromosome synapsis. Reduced expression of these meiosis-related genes is consistent with the meiotic abnormalities observed in stressed mice and may reflect disruption of molecular pathways involved in chromosome synapsis and recombination. Collectively, these findings indicate that adolescent restraint stress is associated with impaired spermatogenesis, altered meiotic marker distribution, abnormal chromosome synapsis, and increased germ cell apoptosis in adult mice.

To our knowledge, this is the first study to systematically characterize the effects of adolescent psychological restraint stress on meiotic progression in male mice. Previous studies primarily focused on sperm parameters, endocrine alterations, or general testicular pathology. By combining spermatogenic stage analysis, meiotic chromosome spread assays, apoptosis assessment, and gene expression profiling, the present study extends current understanding of how psychological stress during a critical developmental period is associated with disturbances in meiotic progression and spermatogenesis.

This study has several limitations that should be acknowledged. The sample size was relatively small, and no formal power analysis was performed. In addition, the restraint stress model may not fully reflect the complexity of psychological stress in humans, and species differences limit direct extrapolation of the findings. In addition, formal RNA integrity assessment (*e.g*., RIN or RQI analysis) and PCR inhibition testing were not performed prior to qPCR analysis. Although all samples were processed using identical procedures, the absence of these quality-control measures should be considered when interpreting the gene expression results. Nevertheless, the consistency of results across different analyses supports the reliability of the conclusions.

## Conclusions

In conclusion, adolescent restraint stress is associated with impaired reproductive organ development and disrupted spermatogenesis in adult male mice. In addition, changes in the expression levels of key meiosis-related genes, including *SPO11*, *DMC1*, *SYCP1*, and *SYCP3*, were observed in stressed mice. Together, these findings suggest that psychological stress during adolescence may have long-lasting detrimental effects on male reproductive function and is associated with alterations in meiotic progression and germ cell survival.

## Supplemental Information

10.7717/peerj.21750/supp-1 Supplemental Information 1 Primer sequences used for qRT-PCR.

10.7717/peerj.21750/supp-2 Supplemental Information 2 Original images of H&E-stained testis, caput epididymis, and cauda epididymis sections, together with TUNEL-stained testicular sections.

10.7717/peerj.21750/supp-3 Supplemental Information 3 Original images of representative spermatocytes at different stages of meiotic prophase I, Periodic Acid–Schiff (PAS)-stained testicular sections, and SYCP3 immunofluorescence staining.

10.7717/peerj.21750/supp-4 Supplemental Information 4 ARRIVE Checklist.

10.7717/peerj.21750/supp-5 Supplemental Information 5 MIQE Checklist.

## Additional Information and Declarations

### Data Availability

The following information was supplied regarding data availability:

The raw measurements are available in the Supplemental Files.
