# Supplementary material for: Adolescent psychological stress impairs spermatogenesis by disrupting meiotic progression in male mice
Source: PeerJ. 2026 Oct 2;14:e21750. doi: 10.7717/peerj.21750 (PMC13637639; doi:10.7717/peerj.21750)
Supplement: Supplementary file 2 [file peerj-14-21750-s001.docx]

**Supplementary Table 1**

To further evaluate whether restraint stress affected the transcriptional levels of meiosis-related genes, the mRNA expression of **Sycp3**, **Sycp1, Spo11**, and **Dmc1** in the testes was examined by qRT-PCR. The primers used are listed in the Table S1.

| **Gene（Sequence accession number）** | **Primer sequence (5′–3′)** | **Product size (bp)** | **Span exons** | **Tm** |
| --- | --- | --- | --- | --- |
| Sycp3  ( NM_011517.2 ) | Forward primer：GGGGCCGGACTGTATTTACT  Reverse primer：AGGCTGATCAACCAAAGGTG | 169 | 1~2 | Forward：59.16  Reverse：58.08 |
| Sycp1  ( NM_011516.2 ) | F：GCGAAGATTGCTTTGGAGAC  R：GCAGATGCCCGCAGATTAT | 296 | 27~30 | Forward：57.47  Reverse：58.06 |
| SPO11  ([NM_012046.3](https://www.ncbi.nlm.nih.gov/entrez/viewer.fcgi?db=nucleotide&id=2419282035)) | F：TGGCCTCTAGTTCTGAGGTTC  R：GAGGTATCATCCGAAGGCCG | 159 | 2~3 | Forward：58.82  Reverse：60.04 |
| Dmc1  ( NM_010059.3) | F：ACAGCTCAACTTCCAGGAACAG  R：GCCACTGAAATCCACTCGAA | 282 | 8~11 | Forward：60.49  Reverse：58.19 |
| β-actin ([NM_007393.5](https://www.ncbi.nlm.nih.gov/entrez/viewer.fcgi?db=nucleotide&id=930945786) ) | F：TGTACCCAGGCATTGCTGAC  R：AACGCAGCTCAGTAACAGTCC | 238 | 5~6 | Forward：60.32  Reverse：60.61 |

Table S1. Primer sequences used for qRT-PCR
